# Effects of the *BDNF* Val66Met Polymorphism and Met Allele Load on Declarative Memory Related Neural Networks

**DOI:** 10.1371/journal.pone.0074133

**Published:** 2013-11-11

**Authors:** Chris M. Dodds, Richard N. Henson, John Suckling, Kamilla W. Miskowiak, Cinly Ooi, Roger Tait, Fruzsina Soltesz, Phil Lawrence, Graham Bentley, Kay Maltby, Andrew Skeggs, Sam R. Miller, Simon McHugh, Edward T. Bullmore, Pradeep J. Nathan

**Affiliations:** 1 Clinical Unit Cambridge, GlaxoSmithKline, United Kingdom; 2 Medical Research Council Cognition and Brain Sciences Unit, Cambridge, United Kingdom; 3 Brain Mapping Unit, Department of Psychiatry, University of Cambridge, United Kingdom; 4 Clinic for Affective Disorders, Department of Psychiatry, Copenhagen University Hospital, Copenhagen, Denmark; 5 School of Psychology and Psychiatry, Monash University, Melbourne, Australia; 6 Cambridgeshire & Peterborough NHS Foundation Trust, Cambridge, United Kingdom; 7 Department of Psychology, University of Exeter, Exeter, United Kingdom; University of Granada, Spain

## Abstract

It has been suggested that the *BDNF* Val66Met polymorphism modulates episodic memory performance via effects on hippocampal neural circuitry. However, fMRI studies have yielded inconsistent results in this respect. Moreover, very few studies have examined the effect of met allele load on activation of memory circuitry. In the present study, we carried out a comprehensive analysis of the effects of the *BDNF* polymorphism on brain responses during episodic memory encoding and retrieval, including an investigation of the effect of met allele load on memory related activation in the medial temporal lobe. In contrast to previous studies, we found no evidence for an effect of *BDNF* genotype or met load during episodic memory encoding. Met allele carriers showed increased activation during successful retrieval in right hippocampus but this was contrast-specific and unaffected by met allele load. These results suggest that the *BDNF* Val66Met polymorphism does not, as previously claimed, exert an observable effect on neural systems underlying *encoding* of new information into episodic memory but may exert a subtle effect on the efficiency with which such information can be retrieved.

## Introduction

Brain-Derived Neurotrophic Factor (BDNF) is a secretory protein that is widely distributed in the human brain with its expression reduced in neurodegenerative disorders including Alzheimer's and Huntington's disease [1]–[5]. The key function of BDNF in the adult brain is to regulate synapse functions including enhancing synaptic transmission, facilitating synaptic plasticity, particularly long-term potentiation (LTP) [6]–[8], and promoting synaptic growth (i.e. synaptogenesis) such as regulating spine density and expression of synaptic proteins [9]–[10]. A genetic variation in the human *BDNF* gene, a single nucleotide polymorphism (SNP) at nucleotide (G196A, rs6265) that converts Valine to Methionine in the pro-domain (codon 66) of BDNF protein, has been identified, with *in vitro* experiments demonstrating that the Met variant is associated with impaired dendritic trafficking of BDNF, segregation into regulated secretory vesicles and synaptic localization, and decreased activity-dependent secretion (18–30% decrease) [11]–[12].

The role of BDNF in modulating LTP has led to much interest in the effect of the *BDNF* Val66Met polymorphism on learning, memory and underlying neural circuitry. Several fMRI studies have found effects of the polymorphism on activation in regions subserving memory, in particular the medial temporal lobe (MTL). However, there is considerable inconsistency surrounding the direction of the effect [13], with some studies finding lower activation in met carriers [14]–[19] and others finding lower activation in val homozygotes [11] [20] [21].

One possible explanation for this inconsistency is that previous studies have included variable numbers of met homozygous subjects in their samples. The met allele is less frequent in the general population than the val allele and therefore the majority of studies have grouped met homozygotes with heterozygotes into a ‘met carrier’ group and compared this with a val homozygote group. If the effect of the polymorphism depends on the number of met alleles then differences across studies in the relative numbers of met homozygous and heterozygous subjects in the ‘met carrier’ group may lead to variability in the effect of the polymorphism on memory related neural activation. Whilst it seems unlikely that this would lead to opposing results across studies, the difficulty of recruiting subjects that are homozygous for the met allele means that the true effect of met allele load on MTL activation remains underexplored.

Conflicting results may also be accounted for by the wide variety of different approaches to controlling for type 1 error adopted by different studies; Whilst some studies have performed a small voxel correction (SVC) for the familywise error (FWE) across the search space e.g. [17] [18], other studies have used uncorrected statistics (e.g. [11] [20]; [14] [19], with alpha levels ranging from 0.05 to 0.001 and extent thresholds (minimum cluster size for significance) ranging from 0 to 10 voxels. The majority of these studies have reduced the number of comparisons carried out by restricting their analyses to regions in the MTL. However, the MTL is a large region which, depending on voxel size and the precise boundary used, may contain in the region of several thousand voxels. Thus, even with an alpha level of 0.001, without a correction for multiple comparisons we would expect to see significant effects in several voxels merely by chance. Moreover, with such a range of statistical approaches, it is perhaps unsurprising that different studies have observed widely different results.

An additional source of inter-study variability may arise from differences in the tasks used to elicit MTL activation. Conflicting genotype results in some studies may be accounted for by the use of nonepisodic memory tasks such as the N-back task, e.g. [11] [15] which often cause deactivation of the hippocampus. Other studies have used episodic memory tasks in which extended periods of encoding or retrieval are contrasted with periods of rest, e.g. [17], making it difficult to rule out confounding effects of nonmemory processes, e.g. attention, on resulting patterns of activation. Of the very few studies that have examined the effects of genotype on activation during *successful* memory encoding and retrieval, results are inconsistent, with some showing no genotype effect [19] and some showing a difference between genotype groups but only with an uncorrected statistical threshold [20]. Moreover, even direct replication attempts using exactly the same tasks as previous studies have produced inconsistent results, e.g. [20], although, as the authors point out, this may be due to differences in behavioural performance between the two studies. Thus, there is at present little consistency in the evidence for a modulatory effect of the *BDNF* Val66Met genotype on neural systems directly linked to episodic memory performance.

In the present study, we attempted to provide a definitive answer to the question of whether the *BDNF* polymorphism affects memory related activation by addressing each of the issues highlighted above. Firstly, we genotyped subjects prior to recruitment in order to attain sufficient numbers of met homozygous subjects to examine the effect of met allele load on activation of memory circuitry. Secondly, for our primary analysis we avoided the multiple comparisons problem by defining regions of interest a priori and averaging BOLD signal across voxels within each region. Finally, we examined effects of the *BDNF* polymorphism on neural activation during *successful* encoding and retrieval to investigate the extent to which any genotype effects are related directly to memory processing. For comparability with previous studies, we examined effects of genotype on a scene encoding and recognition memory task that was adapted from one used in a previous study on the effects of the *BDNF* Val66Met polymorphism on memory related brain activation [17].

## Methods

### Ethics Statement

The study was approved by Cambridge South National Research Ethics Committee (REC reference 11/EE/0360). Participants provided written consent to participate in the study. The consent procedure was approved by the Cambridge South National Research Ethics Committee.

### Subjects

Sixty three healthy subjects (41 males), all right-handed, were recruited for the study from a database of approximately 10,000 subjects with information on the *BDNF* gene polymorphism, held at the Phase I GSK Clinical Unit and the Cambridge BioResource, Cambridge Biomedical Research Centre (CBRC). Of these 63, three subjects withdrew from the study and two subjects' data were excluded from analysis for performance at or close to chance (below 60% overall correct responses at retrieval). Of the remaining 58 subjects, 20 (10 males) were homozygous for the met allele (MetMet), 18 (13 males) were heterozygous (ValMet) and 20 (14 males) were homozygous for the val allele (ValVal). All subjects were Caucasian except one who was of mixed ethnicity. The genotypes in our sample are not in Hardy-Weinberg Equilibrium. This was intentional - we wanted to recruit an equal number of subjects from each of the genotype groups to look at gene-dose effects in a balanced design. The mean age of the subjects was 40 years (range 19–55 years).

Subjects were screened for eligibility to participate. Exclusion criteria included (but were not limited to) the following: any current history of Axis I psychiatric disorders as determined by MINI neuropsychiatric interview; any previous disease or current medical condition, which, as judged by the investigator, could affect the interpretation of data; personal or family history of epilepsy; positive pre-study HIV, Hepatitis B surface antigen or positive Hepatitis C antibody result within 3 months of screening; history of alcohol or substance abuse or dependence in the 6 months prior to screening; regular use of tobacco- or nicotine-containing products within 6 months prior to screening; use of any centrally acting medication; positive urine drug test at screening or when tested at any of the study visits; pregnant females as determined by positive urine hCG test at screening and testing days or lactating females; drug dependence by the DSM-IV criteria within the last 6 months as assessed by the (MINI) interview.

### Procedure

The study was double-blind. Subjects attended a screening session and two testing sessions on separate days. fMRI scanning was performed as part of a wider study examining a variety of neurophysiological and behavioural endpoints, but only the fMRI data are reported here. fMRI scanning was performed on the first testing session. Subjects arrived at the GSK Clinical Unit in the morning and a urine drug screen and alcohol breath test was performed followed by electrophysiological assessment. fMRI scanning was performed between 12.30 and 14.30 hours.

### Episodic Memory Task

The task used in the current study was a picture encoding/retrieval task that was modified from that used in previous studies [17] [22]. The task was composed of an encoding phase and a retrieval phase. During encoding, subjects viewed a single scene on each trial and reported, using one of two keys on a button box, whether the scene was indoor or outdoor. In the retrieval phase, subjects were again shown a single scene on each trial and this time reported, using the same two buttons, whether each scene was old (i.e. they remembered it from the encoding phase) or new. One half of the retrieval trials were old (the other half were new), and only one half of the scenes from encoding were tested. In order to increase the difficulty of the task, ‘new’ scenes were old scenes in which the photograph had been taken from a different viewpoint, and subjects were instructed only to report a scene as ‘old’ if it was exactly the same, i.e. the same scene taken from exactly the same viewpoint.

There was a 10 minute gap between the encoding and retrieval phases during which subjects were instructed to close their eyes and rest without falling asleep. A black screen was presented. After 10 minutes subjects were instructed to open their eyes and advised that the next stage of the task was about to begin. There were 16 encoding blocks and 16 retrieval blocks per phase, each block lasting 24 s, with 20 s blocks of passive fixation (baseline) interleaved. In each block there were 6 trials, leading to 96 encoding trials and 96 retrieval trials in total. Indoor/outdoor and old/new trials were pseudorandomly presented, with the constraint that there were three of each trial type in each block. On each trial, a fixation cross was presented for 1000 ms, followed by the target scene for 3000 ms, during which time the subject was required to make their response.

### fMRI Data Acquisition and Analysis

Subjects were scanned at the Wolfson Brain Imaging Centre in Cambridge, UK. Scanning was performed in a single run including an initial resting state session (not reported here), the encoding phase, the retention interval of further rest, and the retrieval phase. A total of 32 gradient-echo T2*-weighted echo-planar images were acquired per volume, containing blood oxygenation level-dependent (BOLD) contrast. The first six volumes were treated as “dummy” scans and discarded to avoid T1-equilibration effects. The 32 images (slices) contained 64×64 pixels, were positioned at 30° to the anterior commissure–posterior commissure plane, and were 3 mm thick with a 0.5 mm interslice gap. The repetition time between volumes was 2000 ms, with an echo time of 30 ms and 90° flip angle. Data were analyzed using statistical parametric mapping in the SPM8 software (www.fil.ion.ucl.ac.uk). Volumes were realigned (corrected for head motion), slice-time corrected, then spatially normalized to a standard template in MNI space, and spatially smoothed with a Gaussian kernel (8 mm full width at half-maximum). The time series in each session were high-pass filtered (with cutoff frequency 1/120 Hz) and serial autocorrelations were estimated using an AR(1) model.

The fMRI timeseries for each subject were modeled by two, orthogonal approaches. In the “epoch” model, encoding and retrieval blocks were modeled as epochs (boxcar functions) of 24 s and 20 s, convolved with a canonical hemodynamic response function (HRF). This model was used to test neural activity related to general encoding and retrieval states (regardless of memory success for individual trials), as was done in some prior fMRI studies [17] [18] [20]. In the “event” model, on the other hand, each trial within an encoding or retrieval block was modelled as a 3 s boxcar convolved with the canonical HRF, and those trials split into two types (separate regressors): one for stimuli remembered; the other for stimuli forgotten (plus a third type for correct rejections of new items for the retrieval model). This model was used to test for neural activity related specifically to successful encoding or retrieval. This resulted in 4 separate GLMs for each subject:

1. Event encoding model – including separate regressors for subsequent hits and subsequent misses modelled on a trial-by-trial basis.
2. Event retrieval model – including separate regressors for hits, misses and correct rejections modelled on a trial-by-trial basis.
3. Epoch encoding model – including separate regressors for encoding task and rest modelled on a block-by-block basis.
4. Epoch retrieval model – including separate regressors for retrieval task and rest modelled on a block-by-block basis.

These general linear models (GLMs) were fit to every voxel in a least squares sense, i.e, a mass univariate analysis was conducted that resulted in images of parameter estimate (“beta”) in each voxel for each regressor.

Given previous results showing effects of the *BDNF* polymorphism on memory-related activation in the medial temporal lobe (MTL), specifically hippocampus and parahippocampal gyrus, our primary analyses focused on these regions.

Two different second level (group) analyses were carried out – one in which we examined mean activation across ROIs and another in which we examined activation on a voxelwise basis.

#### Group Analysis 1 – Mean activation across voxels

In the first analysis, we defined four structural ROIs using the AAL atlas and marsbar toolbox [23] – left hippocampus, right hippocampus, left parahippocampal gyrus and right parahippocampal gyrus. For each of these regions, individual subjects' mean beta values across all voxels were extracted for the following contrasts using Marsbar, and ANOVAs/t-tests were computed in SPSS.

1. Retrieval: Hits vs correct rejections.
2. Retrieval: Hits vs misses.

The above two contrasts both measure activation related to successful memory retrieval.

1. Encoding: Subsequent hits vs subsequent misses. This contrast reveals activation during encoding trials that predicts subsequent retrieval success.
2. Retrieval blocks vs rest blocks. This contrast reveals regions activated during retrieval task performance relative to a resting baseline and was performed for consistency with previous studies.
3. Encoding blocks vs rest blocks: This contrast reveals regions activated during encoding task performance relative to a resting baseline and was performed for consistency with previous studies.

Effects of group on mean activation in each ROI were examined using repeated measures ANOVAs with ROI as the within-subjects factor and group as the between-subjects factor. Where a significant main effect of group or ROI x group interaction was observed, these were followed up with independent samples t-tests examining effects in each ROI individually.

#### Group Analysis 2 – Voxelwise analysis

In addition to estimating the average activation across voxels within each anatomical ROI, we also performed a voxelwise search for group effects within these regions, using the ROIs to perform a small-volume correction (SVC) of the family-wise error rate (FWE) across the entire mask. ROIs were defined using the AAL atlas and the analysis was carried out using Pickatlas and SPM8. This second, voxelwise approach allows for potential functional inhomogeneity within the MTL (that would be lost by averaging over all voxels), and also provides consistency with some previous studies that performed masked voxelwise analyses across the MTL [11] [17]–[20].

For this second, voxelwise analysis, beta images from each of the four first level GLMs (event encoding, event retrieval, epoch encoding and epoch retrieval) were entered into four separate group GLMs in SPM8, each of which implemented an Analysis of Variance (ANOVA), with the factors group (MetMet, ValMet, ValVal) and memory condition, treating subject as a random effect. Note that each ANOVA assumed a single pooled error [24], whose nonsphericity was estimated by Restricted Maximal Likelihood estimation on data combined across “active” voxels. If these voxels differ only in the scaling of the same error correlation, this pooling of the error is the most sensitive statistical approach. Nonetheless, to allow for the possibility that the error correlation differed across voxels, we repeated the ANOVAs after partitioning the error into separate terms for each ANOVA effect (by constructing contrasts of the data and fitting a separate GLM for each effect in SPM; [24]). There were negligible differences between the pattern of significant results when pooling versus partitioning the error (e.g, no suprathreshold ANOVA effects when partitioning the error that were not also present when pooling the error), so we report the more sensitive pooled error results here.

For both of the group analyses, several planned comparisons were tested. Firstly, in an attempt to replicate previous studies, we combined MetMet and ValMet subjects into a ‘met carrier’ group and compared this with the val homozygous group. Secondly, we compared the met homozygous (MetMet) group with the val homozygous (ValVal) group – this comparison should show the strongest effect if it is met allele load dependent. Where significant group effects were observed in either of the above comparisons, we followed these up with a comparison of the met homozygous (MetMet) group with the heterozygous (ValMet) group in order to examine whether activation was affected by the number of met alleles carried (i.e. effect of met load). The rationale here was firstly to look for an overall effect of the met allele and secondly to examine whether that effect depended on the number of met alleles carried – for this reason, met allele load effects were not explored where no group differences in either of the first two comparisons were observed.

Finally, in order to explore genotype effects in brain regions associated with task performance but outside the MTL, we performed whole brain analyses of task effects across all subjects for successful encoding and retrieval and performed a search across all voxels in these regions to examine group effects, controlling the family-wise error rate (FWE) across the entire brain.

### Behavioural Data Analysis

Behavioural data were subjected to signal detection analysis. Briefly, trials were divided into hits (old scenes correctly labelled as old), misses (old scenes incorrectly labelled as new), correct rejections (new scenes correctly labelled as new) and false alarms (new scenes incorrectly labelled as old) and d prime (d′) was calculated using the formula d′ = z(H)−z(FA), where z(H) is the z-transform of the proportion of hits and z(FA) is the z-transform of the proportion of false alarms.

### Genotyping

DNA was extracted from blood samples via standard methods and genotyped for the *BDNF* Val66Met SNP via TaqMan 50exonuclease assay (Applied Biosystems, Foster City, CA, USA).

## Results

### Demographics

One way ANOVA revealed that the mean age of subjects did not differ significantly between the three genotype groups, F(2,55) = 0.49, p = 0.62. (MetMet M = 41.75 SEM = 2.2, ValMet M = 40.28 SEM = 2.52, ValVal M = 38.45, SEM = 2.51).

Chi-square revealed that there was no significant difference in gender across the three genotype groups, χ^2^(2) = 2.53, p = 0.28.

### Behavioural Results

An independent samples t-test comparing met carriers (MetMet and ValMet) with val homozygotes (ValVal) revealed no significant effect of genotype on d′ (MetMet M = 1.07 SEM = 0.1, ValMet M = 0.97 SEM = 0.08, ValVal M = 1.08 SEM = 0.08, t(57) = −.95, p = .34) or on proportion of hits(MetMet M = 0.59 SEM = 0.03, ValMet M = 0.57 SEM = 0.03, ValVal M = 0.63 SEM = 0.03, t(57) = −1.56, p = .12), or proportion of false alarms, (MetMet M = 0.22 SEM = 0.03, ValMet M = 0.23 SEM = 0.03, ValVal M = 0.24 SEM = 0.02, t(57) = −.26, p = .8). There was also no significant effect of genotype when comparing met homozygotes and val homozygotes: d′, t(38) = −.71, p = .48; percentage of hits, t(38) = −1.5, p = .14; percentage of false alarms, t(38) = −.34, p = .73.

### FMRI Results

Summary statistics for all fMRI and behavioural results are presented in File S1, tables S1 and S2 in File S1.

#### Encoding

**Figure 1:**
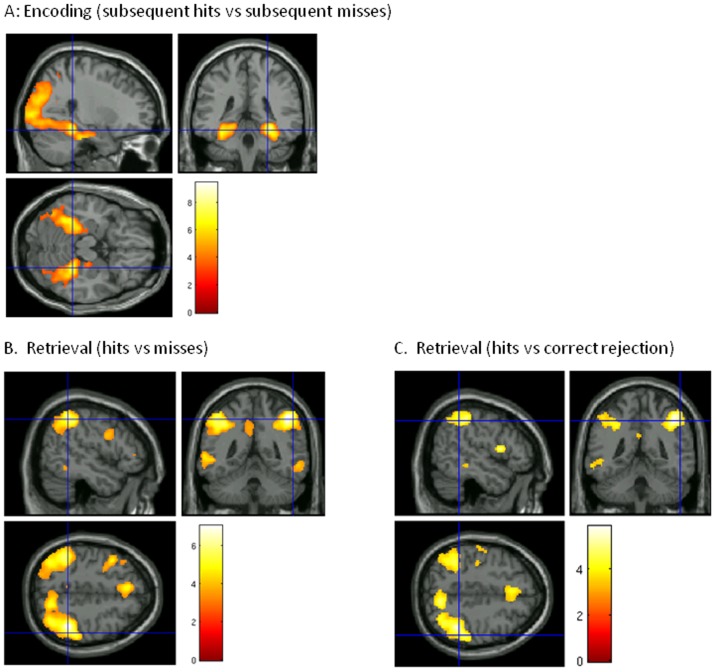
Whole brain task effects: Statistical Parametric Maps overlaid on MNI template brain showing regions that show significant activation during successful encoding (subsequent hits versus subsequent misses, panel A) or successful retrieval (hits versus misses, panel B, or hits versus correct rejections, panel C). SPMs are thresholded at p<0.001 uncorrected for multiple comparisons for display purposes. However, peak voxels survive at FWE p<0.05 corrected for multiple comparisons.

One sample t-test revealed significantly greater activation, averaging across all three groups, during successful encoding (subsequent hits vs subsequent misses) when averaging across all voxels within all 4 anatomical MTL ROIs, t(57) = 2.92, p = .005. Individual one-sample t-tests on average activation across voxels within each ROI confirmed significantly increased activation in all four ROIs: left hippocampus, t(57) = 2.42, p = .02, right hippocampus, t(57) = 2.06, p = .04, left parahippocampal gyrus, t(57) = 2.99, p = 0.004 and right parahippocampal gyrus, t(57) = 3.43, p = 0.001. These ROI effects were broadly confirmed in a whole brain analysis, which showed two large clusters of voxels that showed increased activation for subsequent hits than subsequent misses extending bilaterally from occipital cortex into fusiform gyrus, parahippocampal gyrus and hippocampus (Figure 1A).

Repeated measures ANOVA comparing successful encoding activation in met carriers (MetMet and ValMet) and val homozygotes in the MTL ROIs revealed no main effect of group, F(1,57) = .32, p = .57 and no group x ROI interaction, F(1,57) = 1.16, p = .33. There was also no significant difference between met homozygotes and val homozygotes, F(1,38) = .009, p = .92 and no group x ROI interaction, F(1,38) = .48, p = .69. Voxelwise searches across bilateral hippocampus and parahippocampal gyrus, and across regions activated in the whole-brain task analysis, with a FWE-corrected p-value of 0.05, revealed no voxels that showed a significant effect of group on activation during successful encoding, comparing val homozygotes with either met homozygotes or with met carriers.

Repeated measures ANOVA comparing activation during encoding independent of subsequent retrieval success (encoding blocks – rest blocks) in met carriers and val homozygotes in the MTL ROIs revealed no main effect of group, F(1,57) = 1.08, p = .3 and no group x ROI interaction, F(1,57) = 1.67, p = .18. There was also no significant difference between met homozygotes and val homozygotes, F(1,38) = 1.01, p = .32 and no group x ROI interaction, F(1,38) = .4, p = .75. A voxelwise search across bilateral hippocampus and parahippocampal gyrus with a FWE-corrected p-value of 0.05 also revealed no voxels in which encoding-related activation independent of subsequent success differed significantly by group, when comparing val homozygotes with either met homozygotes or met carriers.

#### Retrieval

Activation averaged across all four MTL ROIs and across all three groups was not significantly greater for hits relative to misses, one sample t-test: t(57) = 1.06, p = .29, or for hits vs correct rejections, one sample t-test: t(57) = 1.16, p = .25. Whole brain analysis of task effects showed a pattern of activation that was similar for hits versus misses and for hits versus correct rejections involving activation in several regions, including bilateral parietal cortex, caudate nucleus, inferior frontal cortex and anterior cingulate cortex (figure 1B+C).

Repeated measures ANOVA comparing hits with misses in met carriers and val homozygotes in the MTL ROIs revealed no main effect of group, F(1,57) = 1.96, p = .17 and no group x ROI interaction, F(1,57) = .13, p = .94. There was also no significant group difference between met homozygotes and val homozygotes, F(1,39) = .19, p = .67, and no ROI x group interaction, F(1,39) = 1.68, p = .18.

**Figure 2:**
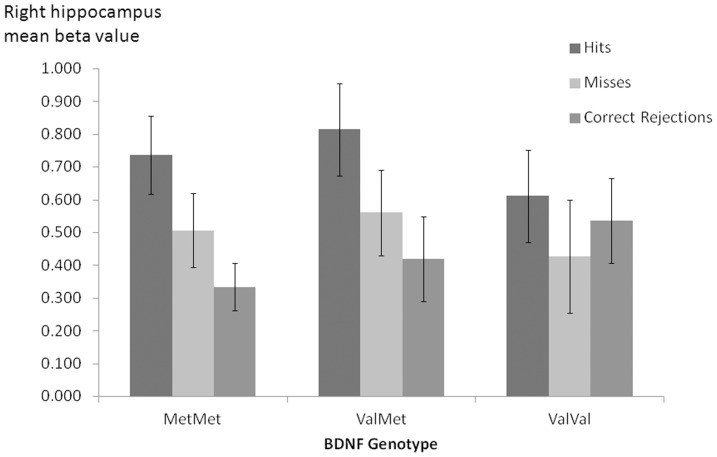
Mean beta values for hits, misses and correct rejections during the retrieval phase in right hippocampus by BDNF Val66Met genotype. Error bars represent SEM.

Repeated measures ANOVA comparing hits with correct rejections in met homozygotes and val homozygotes in the MTL ROIs revealed no significant main effect of group, F(1,57) = 2.12, p = .15 and no significant group x ROI interaction, F(1,57) = 2.47, p = .07. The same ANOVA comparing met carriers and val homozygotes in the MTL ROIs revealed no significant main effect of group, F(1,57) = 2.27, p = .14. However there was a significant group x ROI interaction, F(1,57) = 2.70, p = .048. T-tests examining each ROI individually revealed significantly greater activation in met carriers in the right hippocampus, t(57) = 2.14, p = .04 (Figure 2) but not in the left hippocampus, t(57) = 1.61, p = .11, left parahippocampal gyrus, t(57) = .44, p = .66 or right parahippocampal gyrus, t(57) = 1.58, p = .12. An independent samples t-test comparing successful retrieval related activation (hits-correct rejections) in the right hippocampus in met homozygotes and heterozygotes (i.e. MetMet vs ValMet) revealed no significant effect of the number of met alleles on activation in this region, t(57) = .04, p = .97.

Voxelwise searches across bilateral hippocampus and parahippocampal gyrus, and across regions activated in the whole-brain task analysis, with a FWE-corrected p-value of 0.05, revealed no voxels which showed a significant effect of group on activation during successful retrieval (for either hits versus misses or hits versus correct rejections) comparing val homozygotes with either met homozygotes or with met carriers.

Finally, we also examined the effect of genotype on retrieval-related activation regardless of retrieval success (retrieval blocks vs rest blocks). Repeated measures ANOVA showed no significant difference between met carriers and val homozygotes in the MTL ROIs, F(1,57) = .007, p = .93 and no group x ROI interaction, F(1,57) = .95, p = .42. There was also no significant difference between met homozygotes and val homozygotes, F(1,38) = .15, p = .7 and no group x ROI interaction, F(1,38) = 1.02, p = .38. A voxelwise search across bilateral hippocampus and parahippocampal gyrus revealed no voxels that showed a significant group effect on activation during retrieval regardless of its success that survived p<.05 SVC, FWE-corrected, comparing val homozygotes with either met homozygotes or with met carriers.

### Effect of Age on Memory-Related Activation

Because previous genetic studies have found moderating effects of age on genetic effects on MTL activation, e.g. [25], we repeated all of the fMRI analyses including age as a covariate in the models, and examined effects of group and group x age interactions. Inclusion of the age covariate did not alter any of the non-significant results. However, inclusion of age as a covariate in the ANOVA comparing activation during successful retrieval (hits vs CRs) in met carriers and ValVal subjects resulted in the group x ROI interaction no longer reaching significance, F(1,57) = 2.36, p = .08. A one-way ANOVA including age as a covariate focusing on the right hippocampus also failed to reveal a significant difference between met carriers and ValVal subjects, F(1,57) = 2.36, p = .13.

### Correlation Between Memory Performance and Activation

**Figure 3:**
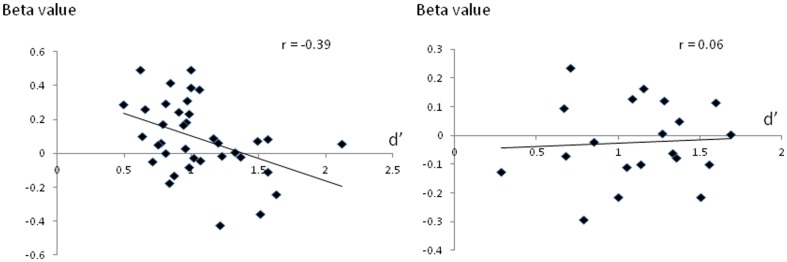
Scatterplots showing correlations between activation during successful retrieval (hits – correct rejections beta value – y axis) in right hippocampus and memory performance (d′ – x axis) in met carriers (left panel) and val homozygotes (right panel).

We examined correlations between activation and memory performance in the right hippocampus as this was the only region that showed a significant effect of group on activation during successful memory retrieval. There was a significant negative correlation between memory performance (d′) and activation during successful memory retrieval (hits – correct rejections beta values) in right hippocampus r = −0.3, p = .02 (figure 3). We also examined correlations separately in met carriers and val homozygotes. There was a significant negative correlation between memory performance and activation in met carriers, r = −.39, p = .01 but not in val homozygotes, r = .06, p = .79. Comparison of the regression slopes of beta values against d′ across genotypes confirmed a significantly stronger correlation in met carriers than in val homozygotes, t(52) = 1.89, p (one-tailed) = .03. Comparison of the regression slopes in met homozygotes and heterozygotes revealed no significant difference in the number of met alleles on the strength of this correlation, t(52) = 0.84, p (one-tailed) = .20.

## Discussion

In the present study, we carried out a comprehensive analysis of the effects of the *BDNF* Val66Met polymorphism on brain activity, as measured by fMRI, during encoding and retrieval of episodic memories. In contrast to previous fMRI studies using very similar paradigms, we found no evidence of differences between *BDNF* genotypes (MetMet, ValMet and ValVal) on encoding-related activation in the hippocampus or parahippocampal gyrus, despite multiple different analyses of the type used in the previous studies. We also found no significant effect of the BNDF Val66Met polymorphism on behavioural performance. In fact, the only group difference that surpassed conventional significance levels concerned activation during retrieval in right hippocampus: Met carriers showed a greater difference in the comparison of correct recognition of studied scenes (hits) versus correct rejection of similar, but unstudied, foil scenes. However, when age was included as a covariate, the difference between met carriers and val homozygotes was no longer significant.

Prior fMRI studies on the effects of the *BDNF* polymorphism on memory-related neural activation have found conflicting results, with some showing decreased activation in met carriers [14]–[19] and others showing increased activation in met carriers, relative to val homozygotes [11] [20] [21]. In the introduction, we outlined three possible explanations for these discrepancies, which we discuss in turn below in light of the present findings.

The first possible explanation we put forward was that previous studies have grouped variable numbers of MetMet and ValMet subjects into a ‘Met carrier’ group, which may lead to variable effects on hippocampal activation if the true effect of the polymorphism is met load dependent. The present results are inconsistent with this account. Although we observed greater activation during successful retrieval in met carriers relative to val homozygotes in the right hippocampus, there was no significant difference in activation for this contrast in this region between subjects who carry one met allele (ValMet) and those who carry two met alleles (MetMet).

Another possible explanation for different results across studies is the use of a variety of different paradigms to elicit MTL activation. Different results across studies may reflect task differences such as the extent to which MTL activation can be directly related to the process of laying down and retrieving information in memory. In the present study, we addressed this issue by performing two analyses – data from each phase (encoding and retrieval) were analysed both on a trial-by-trial basis, revealing MTL activation directly related to successful memory performance, and also on a block-by-block basis to provide consistency with some previous studies, e.g. [17]. For the encoding phase, the method of analysis made no difference to the results – there was no effect of group when we analysed MTL activation on a trial-by-trials basis or on a block-by-block basis. For the retrieval phase, we did observe a group effect when examining MTL activation on a trial-by-trial basis but not on a block-by-block basis. Thus, our results provide partial support for this account, although it should be noted that several previous studies found significant effects of the *BDNF* polymorphism on MTL activation using block designs, which we failed to replicate.

The third possible explanation for discrepancies in results across studies concerns differences in the method used to control for type 1 error (false positives). Prior studies have adopted a variety of approaches to correcting for the multiple comparisons carried out in voxelwise analyses of fMRI data, with some studies performing a small volume correction for the familywise error rate, e.g. [17] [18] and others performing no correction, e.g. [11] [14] [19] [20]. In the present study, we addressed this issue by performing our primary analyses on mean activation across all voxels in each ROI, thereby eliminating the multiple comparisons problem. Using this method, we found no strong evidence for an effect of the *BDNF* polymorphism on MTL activation. The drawback of this method is that it may miss more fine-grained effects in subsets of voxels within each ROI. Therefore, in follow-up analyses we also examined MTL activation on a voxel-by-voxel basis. With the appropriate correction for multiple comparisons, we observed no significant group differences during either encoding or retrieval using this method. In a further exploratory analysis, we also examined differences in activation between genotypes using uncorrected statistics. With an uncorrected threshold of p<0.001, we observed a difference in activation between val and met homozygotes during successful retrieval in four voxels, which, with a search area of approximately four thousand voxels, is precisely the number that we would expect to show a significant difference merely by chance. Thus, our results demonstrate the real possibility of type 1 error occurring in between-group comparisons of BOLD signal when using uncorrected alpha levels.

One solution to the problem of inconsistent results across studies is to perform a meta-analysis in an attempt to identify the true effect of the *BDNF* polymorphism on MTL activation. A recent study did this for all prior fMRI studies on the effects of the *BDNF* polymorphism on hippocampal activation, and found that there was a consistent and relatively large effect size across studies, with met carriers showing reduced hippocampal activation relative to val carriers during memory performance [26]. Unfortunately, however, the meta-analysis itself failed to take into account the multiple comparison problem, by calculating effect sizes from voxels that showed the greatest difference between groups in the original studies, thereby potentially providing greatly inflated effect size estimates (for a full analysis of this problem see [13]). Thus, there is at present no evidence for a consistent effect of the *BDNF* polymorphism on MTL activation across studies.

Although we observed some evidence for group differences in successful retrieval activation when averaging activation across voxels in each ROI, there are several reasons to be cautious in over-interpreting these results. Firstly, the effects were confined to the comparison of hits versus correct rejections, and were not observed in the comparison of hits versus misses. Secondly, the effect of group was confined to the right hippocampus during successful retrieval, and the hippocampus was not strongly associated with performance of this phase of the task. Given the strong link between MTL activation and successful encoding in the present study, any effect of the *BDNF* Val66Met polymorphism on MTL memory circuitry should be observable during the encoding phase. Finally, when we included age as a covariate in the model, the difference between met carriers and val homozygotes in activation during successful retrieval was non-significant, suggesting that at least some of the variance in hippocampal activation apparently due to genetic differences may in fact be driven by age differences. In summary, significant group effects occurred in a single analysis in the context of a wide-ranging investigation of genotype effects and may not be especially robust.

These caveats aside, the present findings are consistent with a subset of prior studies that have found increased hippocampal activation in met carriers [11] [20] [21] [27] which may reflect some form of inefficient processing or compensatory activation. Consistent with this notion, we observed a negative correlation between activation during successful retrieval and memory performance, suggesting that subjects who performed more poorly on the task required greater levels of hippocampal activation. Moreover, this correlation was significantly stronger in met carriers, suggesting that neural inefficiency or compensation in the hippocampus may be more pronounced in this group. An alternative interpretation is that increased activation in met carriers itself represents a marker of impaired neural processing [28], consistent with the view that excess hippocampal activation may contribute to memory impairment and may be associated with widespread degenerative processes in prodromal Alzheimer's disease [29] [30].

Finally, we observed no evidence of differences between *BDNF* genotypes on behavioural memory performance. Many prior studies have examined the effect of the *BDNF* Val66Met polymorphism on episodic memory and some have found impaired performance in met allele carriers [31]–[36]. However, some of the largest studies, that have included several hundred subjects in their samples, e.g. [37]–[39] have failed to replicate the results of these smaller studies, calling into question the robustness of the behavioural effect. Furthermore, a recent meta-analysis of behavioural studies [40] concluded that there was no consistent effect of the polymorphism on any cognitive process. Thus, our findings are consistent with the prior literature in showing no significant effect of the *BDNF* Val66Met polymorphism on episodic memory performance.

It is of course possible that our failure to replicate previous findings reflects a type II error. Possible reasons for a type II error include differences between the present study and previous studies in terms of demographic characteristics such as age or IQ (although our random effects model should account for subject-to-subject variation in such factors) and the fact that the fMRI part of the study was run as part of a wider examination of multiple cognitive and neurophysiological endpoints, which may have led to unusual effects on neurocognitive function. On the other hand, the task we used was clearly suitable for examining group effects on hippocampal activation as we observed highly significant main effects of successful encoding in brain regions that are consistent with many previous fMRI studies of episodic memory. Moreover, we used a comparable or greater sample size relative to previous studies, arguing against the possibility that the study was underpowered.

If the present findings are not due to a type II error, what are the implications for the role of BDNF in hippocampal-based memory processes? Clearly, BDNF plays an important role in synaptic plasticity, as evidenced by preclinical evidence for its ability to modulate LTP. However, the effects of the *BDNF* polymorphism on neural systems underlying episodic memory that are known to depend on synaptic plasticity and LTP are apparently less robust. One possible explanation for this discrepancy is the huge leap in complexity involved in making the transition from in vitro studies to studies of systems level memory systems. There may simply be too many complex factors, such as multiple interacting brain regions, SNPs and psychological processes, that converge at the systems level to mask effects that operate at the level of individual synaptic processes. Alternatively, fMRI may be too blunt a tool to detect such effects, although arguments for the use of neuroimaging in detecting endophenotypes for psychiatric disorders tend to revolve around the claim that imaging methods provide superior sensitivity to such intermediate cognitive biomarkers than do behavioural assays. Moreover, as noted above, the behavioural literature on the cognitive effects of the *BDNF* polymorphism appear to be equally inconsistent.

In summary, we found no significant effect of the *BDNF* polymorphism on hippocampal or parahippocampal activation during memory encoding or on behavioural task performance. Increased activation in right hippocampus during successful retrieval was observed in met carriers relative to val homozygotes suggesting inefficient neural activation, but this was restricted to a specific contrast and analysis, was not dependent on met allele load, and may have been driven by age differences. The present results do not provide strong support for the hypothesis that the *BDNF* Val66Met polymorphism exerts a robust effect on neural circuitry linked to episodic memory.

## Supporting Information

File S1 **Table S1, Mean (SD) beta values for each contrast, group and ROI. Table S2, Mean (SD) percentage of hits, misses, correct rejections, false alarms and d prime for each group.**
(DOCX)

## References

[1] Phillips HS, Hains JM, Armanini M, Laramee GR, Johnson SA, et al (1991) BDNF mRNA is decreased in the hippocampus of individuals with Alzheimer's disease. Neuron 7: 695–702. 1742020 10.1016/0896-6273(91)90273-3

[2] Durany N, Michel T, Kurt J, Cruz-Sánchez F, Cervós-Navarro J, et al (2000) Brain-derived neurotrophic factor and neurotrophin-3 levels in Alzheimer's disease brains. Int J Dev Neurosci 18: 807–813. 11154850

[3] Ferrer I, Goutan E, Marin C, Rey MJ, Ribalta T (2000) Brain-derived neurotrophic factor in Huntington disease. Brain Res 866: 257–61. 10825501 10.1016/s0006-8993(00)02237-x

[4] Hock C, Heese K, Hulette C, Rosenberg C, Otten U (2000) Region-specific neurotrophin imbalances in Alzheimer disease: decreased levels of brain-derived neurotrophic factor and increased levels of nerve growth factor in hippocampus and cortical areas. Arch Neurol 57: 846–51. 10867782 10.1001/archneur.57.6.846

[5] Zuccato C, Ciammola A, Rigamonti D, Leavitt BR, Goffredo D, et al (2001) Loss of huntingtin-mediated BDNF gene transcription in Huntington's disease. Science 293: 493–8. 11408619 10.1126/science.1059581

[6] Figurov A, Pozzo-Miller LD, Olafsson P, Wang T, Lu B (1996) Regulation of synaptic responses to high-frequency stimulation and LTP by neurotrophins in the hippocampus. Nature 381: 706–9. 8649517 10.1038/381706a0

[7] Lu Y, Christian K, Lu B (2008) BDNF: a key regulator for protein synthesis-dependent LTP and long-term memory? Neurobiol Learn Mem 89: 312–323. 17942328 10.1016/j.nlm.2007.08.018 PMC2387254

[8] Ji Y, Lu Y, Yang F, Shen W, Tang TTT, et al (2010) Acute and gradual increases in BDNF concentration elicit distinct signaling and functions in neurons. Nat Neurosci 13: 302–9. 20173744 10.1038/nn.2505 PMC4780419

[9] Tartaglia N, Du J, Tyler WJ, Neale E, Pozzo-Miller L, et al (2001) Protein synthesis-dependent and -independent regulation of hippocampal synapses by brain-derived neurotrophic factor. J Biol Chem 276: 37585–93. 11483592 10.1074/jbc.M101683200

[10] Tyler WJ, Pozzo-Miller L (2003) Miniature synaptic transmission and BDNF modulate dendritic spine growth and form in rat CA1 neurones. J Physiol 553: 497–509. 14500767 10.1113/jphysiol.2003.052639 PMC2343578

[11] Egan MF, Kojima M, Callicott JH, Goldberg TE, Kolachana BS, et al (2003) The BDNF val66met polymorphism affects activity-dependent secretion of BDNF and human memory and hippocampal function. Cell 112: 257–269. 12553913 10.1016/s0092-8674(03)00035-7

[12] Chen ZY, Ieraci A, Teng H, Dall H, Meng CX, et al (2005) Sortilin controls intracellular sorting of brain-derived neurotrophic factor to the regulated secretory pathway. J Neurosci 25: 6156–6166. 15987945 10.1523/JNEUROSCI.1017-05.2005 PMC1201519

[13] Dodds CM, Henson RN, Miller SR, Nathan PJ (2013) Overestimation of the effects of the BDNF val66met polymorphism on episodic memory-related hippocampal function: A critique of a recent meta-analysis. Neuroscience and biobehavioral reviews 37 (4) 739–41. 23348002 10.1016/j.neubiorev.2013.01.019

[14] Banner H, Bhat V, Etchamendy N, Joober R, Bohbot VD (2011) The brain-derived neurotrophic factor Val66Met polymorphism is associated with reduced functional magnetic resonance imaging activity in the hippocampus and increased use of caudate nucleus-dependent strategies in a human virtual navigation task. Eur J Neurosci 33: 968–977. 21255124 10.1111/j.1460-9568.2010.07550.x PMC3084505

[15] Cerasa A, Tongiorgi E, Fera F, Gioia MC, Valentino P, et al (2010) The effects of BDNF Val66Met polymorphism on brain function in controls and patients with multiple sclerosis: an imaging genetic study. Behav Brain Res 207: 377–386. 19874854 10.1016/j.bbr.2009.10.022

[16] Gasic GP, Smoller JW, Perlis RH, Sun M, Lee S, et al (2009) BDNF, relative preference, and reward circuitry responses to emotional communication. Am J Med Genet B Neuropsychiatr Genet 150B: 762–781. 19388013 10.1002/ajmg.b.30944 PMC7891456

[17] Hariri AR, Goldberg TE, Mattay VS, Kolachana BS, Callicott JH, et al (2003) Brain-derived neurotrophic factor val66met polymorphism affects human memory-related hippocampal activity and predicts memory performance. J Neurosci 23: 6690–6694. 12890761 10.1523/JNEUROSCI.23-17-06690.2003 PMC6740735

[18] Hashimoto R, Moriguchi Y, Yamashita F, Mori T, Nemoto K, et al (2008) Dose-dependent effect of the Val66Met polymorphism of the brain-derived neurotrophic factor gene on memory-related hippocampal activity. Neurosci Res 61: 360–367. 18501457 10.1016/j.neures.2008.04.003

[19] Kauppi K, Nilsson LG, Adolfsson R, Lundquist A, Eriksson E, et al (2012) Decreased medial temporal lobe activation in BDNF (66)Met allele carriers during memory encoding. Neuropsychologia 10.1016/j.neuropsychologia.2012.11.028 23211991

[20] Dennis NA, Cabeza R, Need AC, Waters-Metenier S, Goldstein DB, et al (2011) Brain-derived neurotrophic factor val66met polymorphism and hippocampal activation during episodic encoding and retrieval tasks. Hippocampus 21: 980–989. 20865733 10.1002/hipo.20809 PMC3010486

[21] Schofield PR, Williams LM, Paul RH, Gatt JM, Brown K, et al (2009) Disturbances in selective information processing associated with the BDNF Val66Met polymorphism: evidence from cognition, the P300 and fronto-hippocampal systems. Biol Psychol 80: 176–188. 18838100 10.1016/j.biopsycho.2008.09.001

[22] Miskowiak K, O'Sullivan U, Harmer CJ (2007) Erythropoietin enhances hippocampal response during memory retrieval in humans. J Neurosci 27 (11) 2788–92. 17360900 10.1523/JNEUROSCI.5013-06.2007 PMC6672568

[23] Brett M. Anton J, Valabregue R, Poline J (2002) Region of interest analysis using an SPM toolbox [abstract] Presented at the 8th International Conference on Functional Mapping of the Human Brain, June 2–6, 2002, Sendai, Japan. Available on CD-ROM in NeuroImage, Vol 16, No 2.

[24] Henson R, Penny W (2005) ANOVAs and SPM. Imaging 1: 1–24.

[25] Sambataro F, Murty VP, Lemaitre HS, Reed JD, Das S, et al (2010) BNDF modulates normal human hippocampal ageing. Molecular psychiatry 15 (2) 116. 20098437 10.1038/mp.2009.64 PMC3073456

[26] Kambeitz JP, Bhattacharyya S, Kambeitz-Ilankovic LM, Valli I, Collier DA, et al (2012) Effect of BDNF val(66)met polymorphism on declarative memory and its neural substrate: A meta-analysis. Neurosci Biobehav Rev 36: 2165–2177. 22813992 10.1016/j.neubiorev.2012.07.002

[27] Lau JY, Goldman D, Buzas B, Hodgkinson C, Leibenluft E, et al (2010) BDNF gene polymorphism (Val66Met) predicts amygdala and anterior hippocampus responses to emotional faces in anxious and depressed adolescents. Neuroimage 53: 952–961. 19931400 10.1016/j.neuroimage.2009.11.026 PMC2888869

[28] Bakker A, Krauss GL, Albert MS, Speck CL, Jones LR, et al (2012) Reduction of hippocampal hyperactivity improves cognition in amnestic mild cognitive impairment. Neuron 74: 467–474. 22578498 10.1016/j.neuron.2012.03.023 PMC3351697

[29] Putcha D, Brickhouse M, O'Keefe K, Sullivan C, Rentz D, et al (2011) Hippocampal hyperactivation associated with cortical thinning in Alzheimer's disease signature regions in nondemented elderly adults. J Neurosci 31: 17680–17688. 22131428 10.1523/JNEUROSCI.4740-11.2011 PMC3289551

[30] Yassa MA, Stark SM, Bakker A, Albert MS, Gallagher M, et al (2010) High-resolution structural and functional MRI of hippocampal CA3 and dentate gyrus in patients with amnestic Mild Cognitive Impairment. Neuroimage 51: 1242–1252. 20338246 10.1016/j.neuroimage.2010.03.040 PMC2909476

[31] Dempster E, Toulopoulou T, McDonald C, Bramon E, Walshe M, et al (2005) Association between BDNF val66 met genotype and episodic memory. American Journal of Medical Genetics Part B: Neuropsychiatric Genetics 134 (1) 73–75. 10.1002/ajmg.b.30150 15719396

[32] Miyajima F, Ollier W, Mayes A, Jackson A, Thacker N, et al (2008) Brain-derived neurotrophic factor polymorphism Val66Met influences cognitive abilities in the elderly. Genes, Brain and Behavior 7 (4) 411–417. 10.1111/j.1601-183X.2007.00363.x 17973920

[33] Li SC, Chicherio C, Nyberg L, Von Oertzen T, Nagel IE, et al (2010) Ebbinghaus revisited: influences of the BDNF Val66Met polymorphism on backward serial recall are modulated by human aging. Journal of Cognitive Neuroscience 22: 2164–2173. 19925205 10.1162/jocn.2009.21374

[34] Cathomas F, Vogler C, Euler-Sigmund JC, De Quervain DJF, Papassotiropoulos A (2010) Fine-mapping of the brain-derived neurotrophic factor (BDNF) gene supports an association of the Val66Met polymorphism with episodic memory. The international journal of neuropsychopharmacology 13: 975–980. 20482942 10.1017/S1461145710000519

[35] Beste C, Schneider D, Epplen JT, Arning L (2011) The functional BDNF Val66Met polymorphism affects functions of pre-attentive visual sensory memory processes. Neuropharmacology 60: 467–471. 21056046 10.1016/j.neuropharm.2010.10.028

[36] Richter-Schmidinger T, Alexopoulos P, Horn M, Maus S, Reichel M, et al (2011) Influence of brain-derived neurotrophic-factor and apolipoprotein E genetic variants on hippocampal volume and memory performance in healthy young adults. Journal of neural transmission 118: 249–257. 21190051 10.1007/s00702-010-0539-8

[37] Harris SE, Fox H, Wright AF, Hayward C, Starr JM, et al (2006) The brain-derived neurotrophic factor Val66Met polymorphism is associated with age-related change in reasoning skills. Molecular psychiatry 11 (5) 505–513. 16446742 10.1038/sj.mp.4001799

[38] Hansell NK, James MR, Duffy DL, Birley AJ, Luciano M, et al (2007) Effect of the BDNF V166M polymorphism on working memory in healthy adolescents. Genes, Brain and Behavior 6 (3) 260–268. 10.1111/j.1601-183X.2006.00254.x 16848784

[39] Houlihan LM, Harris SE, Luciano M, Gow AJ, Starr JM, et al (2009) Replication study of candidate genes for cognitive abilities: the Lothian Birth Cohort 1936. Genes, Brain and Behavior 8 (2) 238–247. 10.1111/j.1601-183X.2008.00470.x 19077115

[40] Mandelman SD, Grigorenko EL (2012) BDNF Val66Met and cognition: all, none, or some? A meta-analysis of the genetic association. Genes, Brain and Behavior 11 (2) 127–136. 10.1111/j.1601-183X.2011.00738.x PMC3268899 21980924

