# Supplementary material for: Effects of the BDNF Val66Met Polymorphism and Met Allele Load on Declarative Memory Related Neural Networks
Source: PLoS One. 2013 Nov 11;8(11):e74133. doi: 10.1371/journal.pone.0074133 (PMC3823923; doi:10.1371/journal.pone.0074133)
Supplement: File S1 — Table S1, Mean (SD) beta values for each contrast, group and ROI. Table S2, Mean (SD) percentage of hits, misses, correct rejections, false alarms and d prime for each group. (DOCX) [file pone.0074133.s001.docx]

| Contrast | Group | Left Hippocampus | Right Hippocampus | Left Parahippocampal Gyrus | Right Parahippocampal Gyrus |
| --- | --- | --- | --- | --- | --- |
| Encode-Rest | MetMet | 0.17 (0.10) | 0.14 (0.10) | 0.23 (0.14) | 0.26 (0.17) |
|  | ValMet | 0.17 (0.07) | 0.16 (0.09) | 0.22 (0.13) | 0.31 (0.14) |
|  | ValVal | 0.21 (0.18) | 0.18 (0.15) | 0.29 (0.15) | 0.29 (0.17) |
| Encode  Hits-Misses | MetMet | 0.07 (0.24) | 0.04 (0.22) | 0.09 (0.23) | 0.12 (0.31) |
|  | ValMet | 0.12 (0.31) | 0.09 (0.23) | 0.17 (0.28) | 0.20 (0.36) |
|  | ValVal | 0.05 (0.21) | 0.06 (0.20) | 0.06 (0.25) | 0.10 (0.26) |
| Retrieve-Rest | MetMet | 0.14 (0.11) | 0.12 (0.12) | 0.27 (0.22) | 0.28 (0.19) |
|  | ValMet | 0.11 (0.16) | 0.08 (0.15) | 0.21 (0.24) | 0.26 (0.24) |
|  | ValVal | 0.10 (0.22) | 0.13 (0.23) | 0.22 (0.22) | 0.27 (0.25) |
| Retrieve  Hits-Misses | MetMet | 0.09 (0.22) | 0.07 (0.25) | 0.02 (0.33) | 0.06 (0.31) |
|  | ValMet | 0.09 (0.25) | 0.06 (0.24) | 0.05 (0.32) | 0.08 (0.33) |
|  | ValVal | -0.01 (0.18) | -0.02 (0.17) | -0.05 (0.24) | -0.02 (0.21) |
| Retrieve  Hits-Correct Rejections | MetMet | 0.08 (0.22) | 0.09 (0.19) | 0.02 (0.24) | 0.04 (0.22) |
|  | ValMet | 0.06 (0.22) | 0.09 (0.24) | 0.02 (0.24) | 0.04 (0.26) |
|  | ValVal | -0.01 (0.13) | -0.02 (0.14) | 0.00 (0.18) | -0.05 (0.18) |

Table S1

Table S2

|  | % Hits | % Misses | % Correct Rejections | % False Alarms | d prime |
| --- | --- | --- | --- | --- | --- |
| MetMet | 59.2 (11.7) | 43.2 (12.5) | 77.6 (13.9) | 22.4 (13.9) | 1.08 (0.46) |
| ValMet | 56.8 (13.1) | 42.1 (13.1) | 76.8 (12.4) | 23.2 (12.4) | 0.97 (0.32) |
| ValVal | 63.0 (13.4) | 37.1 (11.6) | 75.5 (10.5) | 24.5 (12.5) | 1.08 (0.37) |
